# Redundant functions of I-BAR family members, IRSp53 and IRTKS, are essential for embryonic development

**DOI:** 10.1038/srep40485

**Published:** 2017-01-09

**Authors:** Ai Mei Chou, Kai Ping Sem, Wei Jun Lam, Sohail Ahmed, Chin Yan Lim

**Affiliations:** 1 Neural Stem Cells Laboratory, Institute of Medical Biology, 138648 Singapore; 2 Epithelial Epigenetics and Development Laboratory, Institute of Medical Biology, 138648 Singapore

## Abstract

The insulin receptor substrate of 53 kDa, IRSp53, is an adaptor protein that works with activated GTPases, Cdc42 and Rac, to modulate actin dynamics and generate membrane protrusions in response to cell signaling. Adult mice that lack IRSp53 fail to regulate synaptic plasticity and exhibit hippocampus-associated learning deficiencies. Here, we show that 60% of IRSp53 null embryos die at mid to late gestation, indicating a vital IRSp53 function in embryonic development. We find that IRSp53 KO embryos displayed pleiotropic phenotypes such as developmental delay, oligodactyly and subcutaneous edema, and died of severely impaired cardiac and placental development. We further show that double knockout of IRSp53 and its closest family member, IRTKS, resulted in exacerbated placental abnormalities, particularly in spongiotrophoblast differentiation and development, giving rise to complete embryonic lethality. Hence, our findings demonstrate a hitherto under-appreciated IRSp53 function in embryonic development, and further establish an essential genetic interaction between IRSp53 and IRTKS in placental formation.

Cytoskeletal remodelling to induce plasma membrane and cell shape reorganization in response to signals from neighbouring cells or the environment is essential in cellular activities such as cell migration, phagocytosis and axon pathfinding. This process involves integration of Rho family (Rho, Rac and Cdc42) GTPase activation with the architectural restructuring of actin filaments underlying the plasma membrane to generate membrane protrusions such as filopodia or membrane ruffles. Thus, specialized adaptor proteins that link activated GTPases to the actin meshwork are expected to play important effector and regulatory roles in facilitating cellular responses to various signalling pathways.

The insulin receptor substrate of 53 kDa (IRSp53) is the founding member of a family of adaptor proteins that interacts with GTPases, downstream cytoskeletal effectors, actin filaments and the plasma membrane^1^,^2^,^3^. This family of Inverse-Bin-Amphiphysins-Rvs (I-BAR)- containing proteins consists of five members: IRSp53 (also known as BAIAP2), insulin receptor tyrosine kinase substrate (IRTKS/BAIAP2L1), missing in metastasis (MIM/MTSS1), actin-bundling protein with BAIAP2 homology (ABBA/MTSS1L), and planar intestinal- and kidney- specific BAR domain protein (Pinkbar/BAIAP2L2). As the first to be identified, IRSp53 is the best studied member of the family. It contains three major interaction domains – a N-terminal I-BAR domain, a partial Cdc42/Rac interactive binding (CRIB) domain and a Src homology 3 (SH3) domain. IRSp53 also has 2 other interaction motifs, PDZ and WH2, on its C-terminal region. IRSp53 has been shown in a number of over-expression studies in cultured cells to couple I-BAR domain-induced membrane deformation with signalling pathways by interacting with activated membrane-bound Rho family GTPases at its N-terminus and actin polymerizing/remodeling proteins at its C-terminal domains^1^,^2^,^4^. IRSp53 is known to interact with Rac1 via the I-BAR and with Cdc42 via the CRIB domains to facilitate the formation of membrane ruffles and filopodia^5^,^6^,^7^,^8^,^9^. IRSp53 further interacts, through the SH3 and PDZ domains, with an ever-expanding set of cytoskeletal effectors such as WAVE2, mDia, Mena, Eps8, Shank, dynamin1 and PSD-95^2^,^6^,^8^,^10^,^11^,^12^,^13^,^14^,^15^,^16^,^17^,^18^. This combinatorial interaction and activation mechanism allows IRSp53 to regulate actin and membrane dynamics in wide-ranging cellular processes in different cell types.

Given its central role in modulating cytoskeletal and membrane substructures in response to signals, IRSp53 has been shown to participate in various morphogenetic events and cellular crosstalk such as eye lens formation, myoblast fusion, wound re-epithelialization, dendritic spine formation and synaptic plasticity^15^,^19^,^20^,^21^,^22^,^23^. In particular, IRSp53 knockout (KO) studies have revealed the role of IRSp53 in regulating NMDA receptor-mediated synaptic transmission, leading to impaired cognitive and social behaviours in IRSp53 KO animals^22^,^24^. Although most analyses of IRSp53 function have been focused on its roles in adult tissues and cells, loss of IRSp53 has been shown to result in partial embryonic lethality^22^,^24^, indicating IRSp53 has hitherto uncharacterized functions that are important during embryogenesis.

In this study, we sought to examine the basis of lethality in IRSp53 null embryos, and identified distinct cardiovascular and placental defects as the leading causes of death between embryonic days 10.5 and 18.5. We further explored a potential genetic interaction between IRSp53 and its closest I-BAR family member, IRTKS. We find that loss of both IRSp53 and IRTKS results in complete embryonic lethality, thus revealing their essential compensatory functions in placental development.

## Results

### Knockout of IRSp53 leads to partial embryonic lethality and pleiotrophic phenotypes

**Figure 1:**
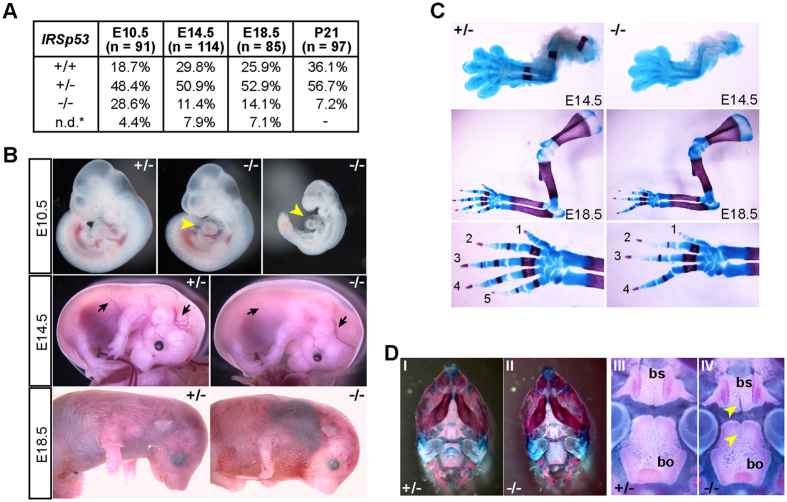
Knockout of IRSp53 leads to partial embryonic lethality and pleiotrophic phenotypes. (**A**) Genotype ratios of heterozygous crosses at different embryonic stages and weaning. 9–13 litters were analysed at each stage. n.d.* resorbed embryos; undetermined genotypes. (**B**) Examples of E10.5, E14.5 and E18.5 IRSp53 heterozygous and KO embryos. Arrowheads point to the pericardium; arrows denote blood vessels. (**C,D**) Alcian blue/Alizarin red staining of E14.5 and E18.5 forelimbs (**C**) and E18.5 skulls (**D**). Panels I, II: ventral views of the skulls. Panels III, IV: higher magnification of the cranial base. Arrowheads: clefts in the basisphenoid (bs) and basioccipital (bo).

In accordance with previous reports, knockout of IRSp53 resulted in partial embryonic lethality, with only a third of IRSp53^*−*/*−*^ animals surviving to adulthood (Fig. 1A, S1A,B). While IRSp53^*−*/*−*^ embryos were obtained at Mendelian ratios from heterozygous intercrosses at embryonic day E10.5, reduced numbers were detected at E14.5, E18.5 and at weaning (Fig. 1A). We found ~50% of KO embryos were lost between E10.5 and E14.5, indicating IRSp53 function is important at these stages. Many of the embryos that survived to E18.5 exhibited developmental defects and a majority of them were dead upon birth or died perinatally such that only 28.8% of KO mice were alive at weaning. These mice did not exhibit obvious phenotypes though they, consistent with prior reports, exhibited neurological deficits in adulthood^22^,^24^,^25^.

To further clarify the requirement for IRSp53 during embryonic development, we characterized the phenotypes of IRSp53^*−*/*−*^ embryos at E10.5, E14.5 and E18.5. At E10.5, the majority of the KO embryos were phenotypically comparable to their wild-type and heterozygous littermates (Fig. 1B). However, 21% (n = 24) were developmentally delayed and exhibited cardiac abnormalities and pericardial edema (Fig. 1B). At E14.5, the KO embryos showed pleiotropic phenotypes, including small size (n = 14/31), subcutaneous edema (n = 15/31), reduced vascular branching, and skeletal malformations (n = 14/31) (Fig. 1B). At E18.5, most of the IRSp53 null embryos were smaller (n = 7/12) and 25% of these embryos exhibited severe subcutaneous haemorrhages and skeletal defects (Fig. 1B). To further examine the skeletal defects observed in E14.5 and E18.5 IRSp53KO embryos, we stained the embryonic skeletons with Alcian blue and Alizarin red. The most overt phenotype was characterized by forepaw oligodactyly, particularly the lack of digit V, while carpal and long bone formation appeared normal (Fig. 1C). We also observed pronounced clefts in the basisphenoid and basioccipital bones in the cranial base of IRSp53^*−*/*−*^ embryos. (Fig. 1D). Together, our results demonstrate that IRSp53 function is indeed vital for normal development and loss of its function adversely affects multiple morphogenetic events in the embryos.

### IRSp53 deletion leads to defective heart development

**Figure 2:**
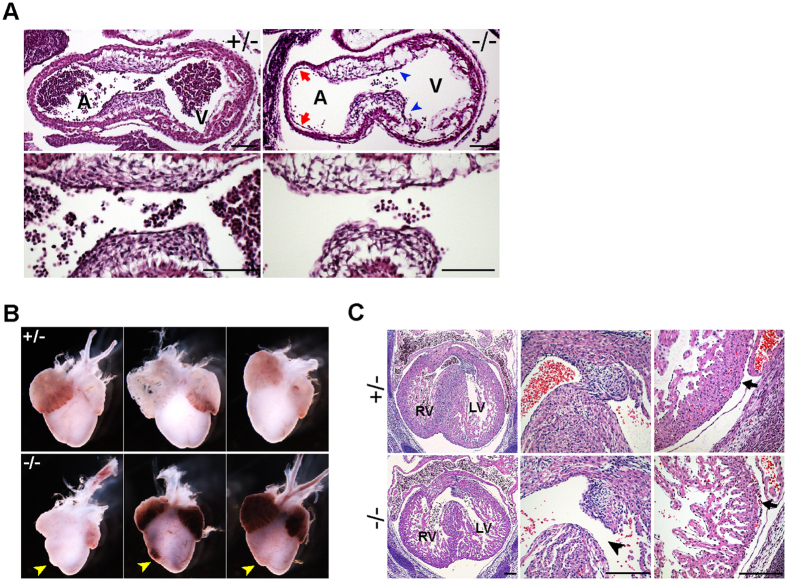
IRSp53 deletion results in defective cardiac development. (**A**) Histological analysis of sagittal sections of E10.5 embryonic heart in IRSp53^+/−^ and IRSp53^*−*/*−*^ embryos. Lower panels show the atrioventricular (AV) cushions at higher magnification. Hypocellular AV cushions (arrowheads) and detachment of endocardium from the myocardium (arrows) were observed in the IRSp53^*−*/*−*^ embryos. A, atrium, V, ventricle. Scale: 100 μm. (**B**) E14.5 hearts dissected from heterozygous and null embryos. Misshapened right ventricles were noted in IRSp53^*−*/*−*^ hearts (arrowheads). (**C**) Histology of E14.5 heterozygous and null hearts. H&E of transverse heart sections (left panels) revealed defects in the interventricular septum (middle panels, arrowhead), thin ventricular walls (right panels) and detachment of the epicardium from the myocardium (arrow) in IRSp53^*−*/*−*^ embryos. RV, LV: right, left ventricle. Scale: 200 μm.

Mid-gestational lethality is often associated with abnormalities in cardiovascular and placental development. While we did not observe strong IRSp53 expression in the developing hearts of E8.5 and E10.5 embryos, early cardiac defects were evident (Fig. S1C,D). At E10.5, histological examination revealed the atrioventricular endocardial cushions in IRSp53^*−*/*−*^ hearts were poorly developed, with distinctly fewer mesenchymal cells compared to that in heterozygous controls (Fig. 2A). In addition, the endocardium, which is closely apposed to the myocardium in controls, appeared to be largely detached in the IRSp53 null (Fig. 2A). At E14.5, the gross appearance of atria chambers of IRSp53^*−*/*−*^ hearts was normal, but the right ventricles were smaller and misshapen (Fig. 2B). Upon histological analysis, we further found interventricular septum (IVS) defects that were consistent with aberrant cardiac cushion formation and maturation in the IRSp53^*−*/*−*^ hearts (Fig. 2C). In all three mutants analyzed, the mesenchymal cushions at the apex of the IVS were smaller and the septa were not closed by E14.5, compared to wildtype and heterozygous littermates (Fig. 2C). The mutants also displayed thinner, hypocellular ventricular walls and IVS with diffuse hyper-trabeculation and non-compaction of myocardial fibres, in contrast to the well-developed and compact ventricular walls and IVS in controls. In addition, we observed separation of the ventricular epicardium from the myocardium with red blood cells in the subepicardial space in the mutants but not in control embryos (Fig. 2C). Our results thus indicate IRSp53 plays an important role in early cardiac development, and heart abnormalities resulting from its loss are likely to be a major cause of lethality of IRSp53^*−*/*−*^ embryos.

### Loss of IRSp53 affects placental morphology and function

**Figure 3:**
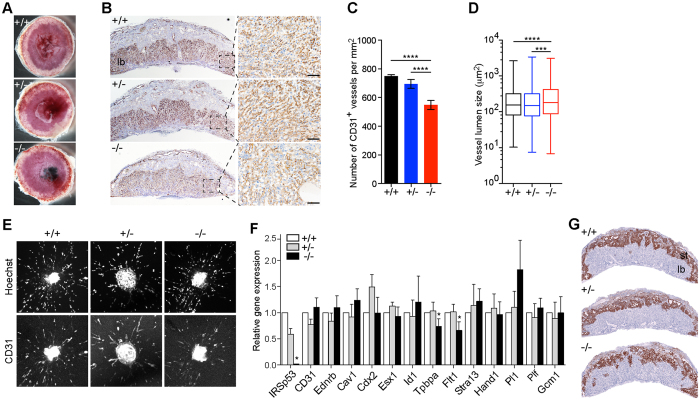
Loss of IRSp53 affects placental morphology and function. (**A**) Morphology of wildtype, heterozygous and null placentas at E14.5. Haemorrhages were observed in the IRSp53 KO. (**B**) Labyrinth vasculature revealed by immunostaining for CD31 in E14.5 placentas. Right panels show boxed areas at higher magnification. Scale: 100 μm. (**C**) Number of CD31^+^ vessels per unit area in E14.5 placentas. Three placentas were analysed for each genotype. Error bars = s.d. ****p < 0.0001, two-tailed *t*-test. (**D**) Lumen size of CD31^+^ blood vessels in E14.5 placentas. Whiskers mark maximum and minimum values. Three placentas were analysed for each genotype. ***p < 0.001, ****p < 0.0001, two-tailed *t*-test. (**E**) *In vitro* microvessel formation from E14.5 umbilical arterial explants examined by staining explants cultured for 3 days in collagen I gels with Hoeschst and CD31 antibodies. (**F**) Expression of lineage marker genes in E14.5 placentas measured by qRT-PCR. Values normalized to *Tbp*, plotted relative to WT. Error bars = s.d. of 4 independent sets of placentas, *p < 0.05, paired *t*-test. (**G**) In situ hybridization of *Tpbpa* transcripts to examine the spongiotrophoblast layer of E14.5 placentas. st, spongiotrophoblast layer; lb, labyrinth layer.

In addition to heart defects, we observed phenotypes in IRSp53 nulls that were consistent with placenta abnormalities, such as severe subcutaneous edema and placenta haemorrhages (Figs 1B and 3A). These and finding IRSp53 to be highly expressed in the embryonic placenta (Fig. S2A), led us to investigate how loss of IRSp53 may affect placental development. To examine effects on labyrinth vasculature, we stained sections of E14.5 placenta for the endothelial cell marker, CD31 and found the vascularization of IRSp53^*−*/*−*^ placentas was less dense with markedly decreased CD31^+^ staining compared to controls (Fig. 3B). While the labyrinth thickness was unaltered, IRSp53KO placentas were characterized by significantly reduced vasculogenesis, with 27% fewer blood vessels per unit area, corresponding to a 31.1% increase in the average vessel size compared to wild-type (Fig. 3C,D, S2B). As deletion of *Cdc42*, the IRSp53-interacting GTPase, was previously shown to impair blood vessel formation, sprouting and remodelling^26^, we sought to examine the effects of IRSp53 loss on angiogenesis. To this end, we tested the vascularization capacity of umbilical arterial explants harvested from E14.5 embryos when cultured in VEGF-containing media. We found no significant differences in the ability of cells in umbilical arterial explants from control or IRSp53KO embryos to migrate and form CD31^+^ microvessels in collagen gel cultures (Fig. 3E), suggesting that the angiogenic response to growth factors is retained in the absence of IRSp53 function.

Our results suggest loss of IRSp53, unlike Cdc42, does not affect endothelial cell migration and branching morphogenesis in a cell-autonomous manner. We thus hypothesized that decreased vasculogenesis in the labyrinth of IRSp53^*−*/*−*^ placentas resulted from deficiencies in trophoblast development, which is required to provide morphogenetic signals and structural support for normal vascularization^27^. To examine this, we first analysed endothelial and trophoblast cell markers by qPCR^28^, and found that while markers of endothelial function, *Pecam1, Ednrb, Cav1*, were unchanged, the expression of spongiotrophoblast markers, *Tpbpa* and *Flt1*, were reduced to ~70% of wild-type levels in the IRSp53^*−*/*−*^ placentas (Fig. 3F). The trophoblast marker *Cdx2*, labyrinth syncytiotrophoblast markers *Esx1, Id1*, and *Gcm1* were not significantly changed. The expression of markers of the different types of trophoblast giant cells (TGC) including *Stra13, Hand1, Plf* and *Pl1* were also not significantly altered (Fig. 3F). Consistent with these gene expression changes, we found the spongiotrophoblast layer in IRSp53KO placentas to be thinner, highly disorganized, and contained reduced *Tpbpa*-positive cells as revealed by *in situ* hybridization, compared to controls (Fig. 3G). By contrast, alkaline phosphatase staining of the labyrinth, which marks the trophoblast cells lining the maternal sinusoids, revealed no major alterations in the organization of maternal blood spaces and labyrinth trophoblast differentiation in the IRSp53KO placentas (Fig. S2C). These results point to defective trophoblast development and differentiation, primarily of the spongiotrophoblast lineage, in the absence of embryonic IRSp53. Thus, our findings indicate that IRSp53 function is vital to support the growth and function of spongiotrophoblast cells in the junctional layer of the placenta but, unlike its binding partner Cdc42, does not directly affect angiogenesis *in vitro*.

### IRTKS functions with IRSp53 to mediate normal embryonic and placenta development

**Figure 4:**
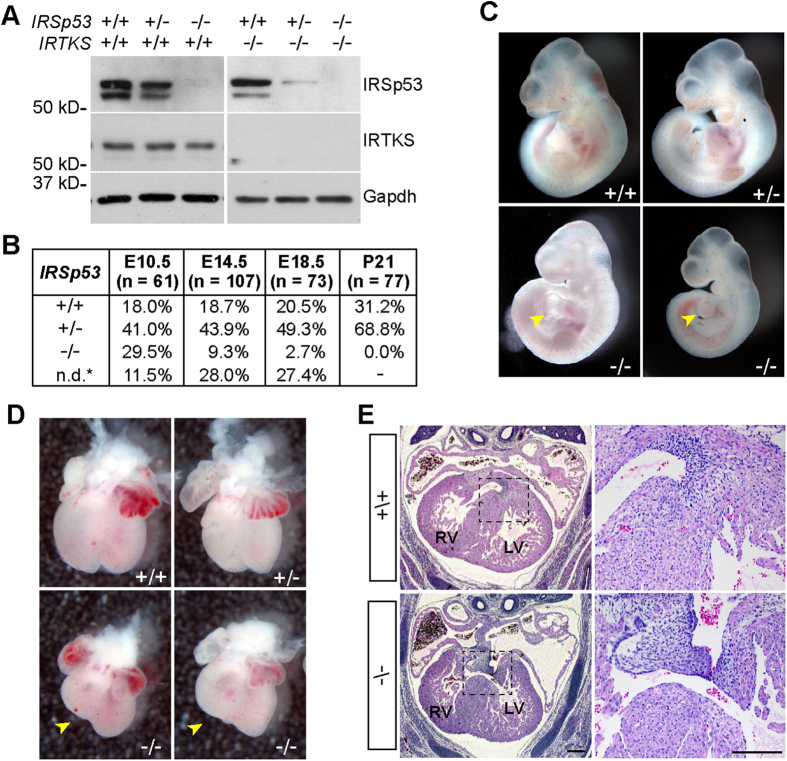
IRTKS and IRSp53 double knockout leads to embryonic lethality. (**A**) Western blot of IRSp53 and IRTKS in total protein lysates of embryonic fibroblasts. The images were cropped for clarity of presentation. Full-length blots are presented in supplementary figure S5A. (**B**) Genotype ratios of IRSp53^+/−^; IRTKS^*−*/*−*^ intercrosses at different embryonic stages and weaning. 7–13 litters were analysed for each stage. n.d.* resorbed embryos; undetermined genotypes. (**C**) Examples of E10.5 IRTKS^*−*/*−*^ embryos that are wild-type, heterozygous or null for IRSp53. Arrowheads point to heart malformations in the double knockout (dKO) embryos. (**D**) Embryonic hearts dissected at E14.5. Misshapen right ventricles were observed in the dKO hearts (arrowheads). (**E**) Histological analysis of embryonic hearts at E14.5 in IRSp53^+/+^; IRTKS^*−*/*−*^ and IRSp53^*−*/*−*^; IRTKS^*−*/*−*^ embryos. H&E staining of transverse sections (left panels) revealed abnormalities in the membraneous ventricular septum (boxed) of dKO hearts, similar to phenotype observed in IRSp53^*−*/*−*^ single mutants. Right panels show boxed region at higher magnification. Scale: 200 μm.

Since only half of *IRSp53*^*−*/*−*^ embryos died in mid-gestation despite IRSp53 functions in cardiac and placental development, we decided to explore whether there was redundancy of IRSp53 with other I-BAR domain proteins. To address this, we generated an insulin receptor tyrosine kinase substrate (IRTKS) knockout mouse (Fig. 4A, S3). IRTKS shares 42% sequence identity with IRSp53 and is the closest member in the I-BAR domain family to IRSp53 (Fig. S3)^29^. Similar to IRSp53, IRTKS is phosphorylated upon insulin stimulation and its over-expression in cells leads to actin-rich membrane protrusions^3^,^30^. Notably, IRTKS lacks a Cdc42-interacting domain, but its I-BAR and SH3 domains have been shown to interact with several known IRSp53 partners such as Rac, Eps8 and Shank, suggesting the two proteins may overlap in mediating actin cytoskeletal remodelling induced by growth factor signalling^30^,^31^,^32^,^33^.

Consistent with previous reports, IRTKS^*−*/*−*^ mice did not exhibit developmental problems and were phenotypically normal (data not shown)^34^. To examine whether IRTKS acts redundantly with IRSp53 during embryogenesis, we generated mice heterozygous for IRSp53 in the IRTKS null background, and analysed embryonic litters from IRSp53^+/−^;IRTKS^*−*/*−*^ intercrosses (Fig. 4A,B). While we obtained similar genotype ratios at E10.5 and E14.5 from intercrosses of *IRSp53*^+/−^ or *IRSp53*^+/−^;*IRTKS*^*−*/*−*^ mice, deletion of both IRSp53 and IRTKS resulted in significantly reduced viability (2.7%) at E18.5 compared to IRSp53 null alone (14.1%). Notably, of the ten litters we analyzed at E18.5, we found non-resorbed embryos in only 1 litter. We also did not detect any escapees from the 13 litters analysed at weaning (Fig. 4B). Thus, our results show that double knockout (dKO) of IRSp53 and IRTKS leads to complete embryonic or perinatal lethality, suggesting IRTKS functionally overlaps with IRSp53 in processes critical for survival.

To clarify the processes in which IRSp53 and IRTKS may have redundant roles, we first examined cardiac development in the dKO embryos. At E10.5, we found that dKO embryos exhibited heart tube malformation and pericardial edema with similar ratios as single IRSp53 null embryos at the same stage (Figs 4C and 1D). At E14.5, we found that the dKO hearts also displayed misshapen right ventricle morphology and incomplete IVS closure and malformation of the mesenchymal membranous part of the septa (Fig. 4D,E), but these phenotypes were not significantly more severe than that observed in IRSp53 null embryos. Thus, concurrent deletion of IRTKS did not exacerbate the early heart defects observed in IRSp53^*−*/*−*^ embryos, indicating that IRTKS does not function in place of IRSp53 in cardiac development at these stages.

**Figure 5:**
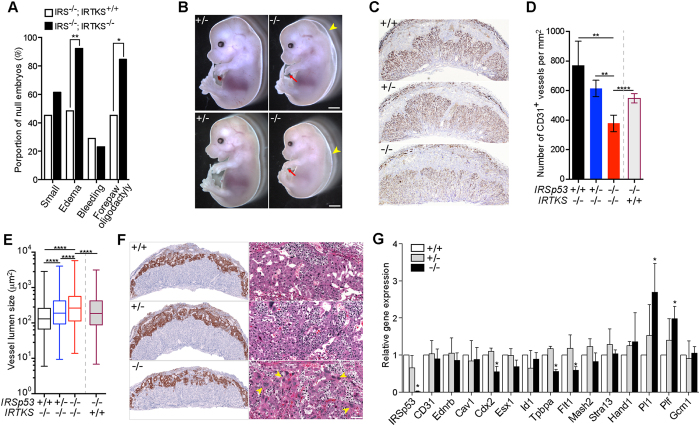
IRTKS functionally overlaps with IRSp53 in placental development. (**A**) Analysis of overt phenotypes in E14.5 IRSp53 knockout embryos in IRTKS^+/+^ (n = 31) or IRTKS^*−*/*−*^ (n = 13) background. *p < 0.05, **p < 0.01, z-score test for two population proportions. (**B**) E14.5 IRSp53^+/−^; IRTKS^*−*/*−*^ and IRSp53^*−*/*−*^; IRTKS^*−*/*−*^ embryos. Arrowheads point to the severe subcutaneous edema and arrows point to the lack of posterior digits in the right forepaw of the dKO mutants. Scale: 0.5 mm. (**C**) Labyrinth vasculature revealed by CD31 immunostaining in E14.5 placentas. (**D**) Number of CD31^+^ vessels per unit area in E14.5 placentas. Three placentas were analysed for each genotype. The IRSp53^*−*/*−*^;IRTKS^+/+^ results included for comparison. Error bars = s.d. **p < 0.01, ****p < 0.0001, two-tailed *t*-test. (**E**) Measurement of lumen size of CD31^+^ blood vessels in E14.5 placentas. Whiskers mark maximum and minimum values. Three placentas were analysed for each genotype. The IRSp53^*−*/*−*^;IRTKS^+/+^ results included for comparison. ****p < 0.0001, two-tailed *t*-test. (**F**) In situ hybridization for *Tpbpa* reveal spongiotrophoblast organization in E14.5 placentas of IRSp53 +/+, +/− and −/− in IRTKS^*−*/*−*^ background. Right panels show the spongiotrophoblast layer in H&E sections at higher magnification. Patches of trophoblast giant cells (yellow arrowheads) were observed in the spongiotrophoblast layer of double KO placentas. Scale: 100 μm. (**G**) Expression of marker genes in E14.5 placentas measured by qPCR. Values normalized to *Tbp*, plotted relative to IRSp53^+/+^;IRTKS^*−*/*−*^ placenta. Error bars = s.d. of 3 independent sets of placentas, *p < 0.05 paired *t*-test.

At E14.5, the most significantly aggravated phenotypes observed when IRTKS was deleted in addition to IRSp53 were subcutaneous edema and oligodactyly (Fig. 5A). Almost all dKO embryos exhibited severe subcutaneous edema (14/15) and oligodactyly (13/15) (Fig. 5A,B), leading us to hypothesize that loss of IRSp53 and IRTKS led to increased embryonic lethality due to exacerbated placental defects. Indeed, while IRTKS deletion alone had no overt impact on the placenta, we found loss of both IRTKS and IRSp53 led to further reduction of CD31^+^ vasculature compared to IRSp53 KO (Fig. 5C). We observed that wild-type and IRTKS^*-/-*^ placentas exhibited similar number of CD31^+^ vessels but vessel density of dKO mutants was only ~50% of wild-type and, more significantly, 30% less than that in IRSp53 KO placentas (Fig. 5D). We also found the mean vessel size to be ~12.4% larger in the double mutants compared to IRSp53^*−*/*−*^ alone (Fig. 5E), indicating that loss of both IRTKS and IRSp53 led to a more severe impairment of labyrinth vascularization than single IRSp53 KO. As was the case with IRSp53, we found that double deletion of IRTKS and IRSp53 did not affect *in vitro* angiogenesis of umbilical arterial explants in response to VEGF signals (Fig. S4).

We observed, however, that dKO placentas displayed much thinner and significantly more disorganized *Tpbpa*^+^ spongiotrophoblast layer compared to IRSp53^*−*/*−*^ placentas (Fig. 5F). In addition, we found that while parietal TGCs line the outermost part of the spongiotrohoblast layer in control placentas, patches of giant cells were interspersed with the spongioblasts cells in the dKO placentas (Fig. 5F). Studies have shown that trophoblast differentiation to TGCs involves extensive changes in cytoskeletal and membrane reorganization^35^. We thus hypothesized that trophoblast differentiation is more severely impacted by the combinatorial loss of IRSp53 and IRTKS, resulting in significantly reduced placenta function that fail to support late gestational development of dKO embryos. To characterize potential changes in trophoblast lineages, we carried out qPCR analyses of lineage markers and found that the expression of *Tpbpa* and *Flt1* were significantly decreased by >40% in the dKO mutants (Fig. 5G). We also observed marked decrease in the levels of trophoblast marker *Cdx2*, while labyrinth trophoblast marker *Gcm1,* was unchanged in the dKO placentas. Examination of labyrinth trophoblasts by alkaline phosphatase staining also did not reveal significant defects in the differentiation and organization of these cells (Fig. S4B). Most notably, we detected significantly increased levels of TGC markers *Pl1* and *Plf*, consistent with the large patches of giant cells observed in the junctional zone of the dKO placentas (Fig. 5G). These results show that while spongiotrophoblast differentiation was significantly constrained in the dKO mutants, differentiation to parietal, spiral artery-associated, and canal TGCs was not adversely impacted, and may instead be amplified at the expense of spongiotrophoblast differentiation. Our results thus indicate that spongiotrophoblast differentiation and the function of the junctional zone was more severely compromised in the dKO mutants, resulting in the increased lethality of these embryos and point to a functional redundancy between IRSp53 and IRTKs in supporting placenta development.

## Discussion

The I-BAR domain proteins belong to the superfamily of BAR domain proteins, which also includes the F-BAR and N-BAR families. These proteins function in coordinating interactions between signal transducers, actin cytoskeleton and membrane components to effect changes in membrane curvature necessary for processes such as cell-cell communication, phagocytosis, angiogenesis and cell migration. Despite their involvement in physiological processes essential for embryonic development, loss-of-function studies of individual I-BAR proteins revealed pathophysiological effects that manifest primarily in adult tissues. For instance, MIM was shown to be dispensable for embryonic development but is required for maintaining the integrity of kidney epithelia intercellular junctions, B-cell development, and glutamatergic synaptic transmission^36^,^37^,^38^. In addition, we, in this study, and others have found that IRTKS deficient mice, while defective in insulin regulation, exhibit no developmental defects^34^. To date, IRSp53 is the only I-BAR member shown to exhibit altered Mendalian ratios at birth when deleted in mice; however, its role during embryonic development was not investigated^22^,^24^.

The discordance between the importance of cellular processes mediated by I-BAR proteins and the lack of overt embryonic phenotypes in gene knockout models suggest significant functional redundancy exist within I-BAR family members. In this study, we find that IRSp53 function is vital for cardiac and placental development and loss of IRSp53 results in lethality of >50% of null embryos at mid- to late- gestation. Our findings further reveal for the first time, a key genetic interaction between IRSp53 and its closest family member, IRTKS, in trophoblast differentiation and placental formation that results in complete embryonic lethality when both genes are inactivated. IRTKS is co-expressed with IRSp53 in various embryonic tissues, such as the cartilage primordium of nasal septum and coccyx, segmental bronchi in the lung, primitive glomeruli in the kidney, epidermis of the skin and spongiotrophoblast layer in the placenta (our unpublished data). However, our findings are consistent with redundancy between IRSp53 and IRTKS in placental development but not in the formation of the heart and other tissues in which the proteins are co-expressed. It is possible that other members of the I-BAR family functionally compensate for the loss of IRSp53 in those tissues. Of note, IRSp53, MIM and Pinkbar have all been found to be expressed in the polarized epithelial cells of adult and embryonic kidney^37^,^39^. To fully understand the functional interplay amongst the I-BAR family members in different tissues and developmental processes, the generation and analysis of combinatorial knockout mice will be crucial.

Our study shows that normal cardiac development is affected in the absence of IRSp53. Distinct hypoplasia of the AV endocardial cushions and the mesenchymal portion of the IVS found in developing IRSp53 null hearts alludes to possible defects in epithelial-to-mesenchymal transition (EMT) of the endocardium cells and the subsequent mesenchymal proliferation during valve formation^40^,^41^. Interestingly, RhoA-mediated signaling, but not Rac1 or Cdc42, have been implicated in EMT of avian endocardial cells^42^. It remains to be examined whether IRSp53 is indeed involved in EMT at AV cushions, and if so, whether it is acting with or independently of Rac1 and/or Cdc42. In addition, our findings have revealed a functional requirement for IRSp53 in AV cushions development, ventricular trabeculation and myocardium growth and maturation. These processes are highly regulated by reciprocal signaling during the development and growth of the endocardium, epicardium and myocardium^40^,^43^,^44^. Studies have shown that actin reorganization facilitating the rapid assembly of adherens junctions between apposing filopodial tips that extend from migrating cells, helps to establish intercellular adhesions between embryonic epithelial cells during development and in culture^45^,^46^,^47^,^48^,^49^. Furthermore, activation of the Cdc42-VASP/Mena pathway, which IRSp53 is part of, is implicated in the cell-cell adhesion processes^47^,^48^. We speculate that Cdc42-IRSp53-VASP/Mena mediated cytoskeletal remodeling and filopodia formation also plays a role in establishing cellular adhesion and cell-matrix interactions within the endocardial and epicardial epithelia. Following this model, loss of IRSp53 would adversely impact formation of the endocardial and epicardial cell layers and their interaction with the myocardium, thereby disrupting the complex cross-talk between these cells necessary for cardiac development.

IRSp53 and IRTKS are central modulators of actin dynamics downstream of the Rho family of small GTPases Cdc42, Rac1 and Rif^2^,^8^,^10^,^33^. Consistent with their activities in mediating major physiological functions through interactions with multiple partners in different pathways, knockout models of Cdc42 and Rac are embryonic lethal at early gestation stages^50^,^51^. While the cardiac phenotypes of IRSp53 mutants suggest that IRSp53 functions, at least in part, within the Cdc42-VASP and/or Mena pathway during embryonic heart development, IRSp53 and IRSp53/IRTKS dKO embryos also exhibited phenotypes that do not correspond with reported Cdc42- and Rac1- deficiencies. These findings suggest that IRSp53 and IRTKS potentially partner with other signal transducers to mediate their functions in different cells and developmental processes. Notably, IRSp53 single KO and IRSp53;IRTKS dKO embryos displayed a forepaw oligodactyly phenotype, which is most similar to the loss-of-function of *Lmbr1*, a membrane protein involved in sonic hedgehog signalling during limb patterning^52^. Specific loss of posterior digits has also been reported in *Blimp1* mutants that fail to regulate signalling in the posterior mesenchyme of developing forelimbs^53^. By contrast, inactivation of *Cdc42* or *Rac1* in the limb bud mesenchyme, mediated by Prx1-cre, was shown to result in syndactyly in fore- and hind- limbs, as well as shortening of long bones in the limbs and skeleton^54^,^55^. Inactivation of IRSp53 and IRTKS also did not significantly affect neovessel formation of umbilical arterial explants in response to VEGF signaling *in vitro*, in contrast to the defective embryonic vasculogenesis and sprouting angiogenesis observed in Cdc42- or Rac1- inactivated vascular endothelia^26^,^56^,^57^,^58^,^59^. Our study further indicates that IRSp53 and IRTKS functions are essential for proper trophoblast differentiation and organization, particularly of the spongiotrophoblast layer, a phenotype that has not been reported for Cdc42 and Rac1 mutants. Hence, it is likely that IRSp53 and IRTKS play vital roles in mediating both Cdc42- and Rac1- dependent as well as independent pathways during the morphogenesis and development of different organs and tissues in the embryo. Additional experiments will be necessary to elucidate new players in these pathways.

In conclusion, we have illustrated the importance of IRSp53-mediated pathways in embryonic morphogenesis and uncovered a novel, indispensable genetic interaction between IRSp53 and its closest family member, IRTKS. Our findings point to complex redundancy amongst BAR domain family members that function within different signaling pathways to effect the normal development of different embryonic tissues and organs. Future elucidation of these intricate genetic interactions will be necessary to clarify the critical mechanisms that control cell signaling and morphogenesis during mammalian embryonic development.

## Materials and Methods

### Generation of IRSp53 and IRTKS knockout mice and husbandry

IRSp53 knockout mice were derived from ES cell line XG757 (BayGenomics, CA, USA) and backcrossed for 10 generations into C57BL/6J background. For IRTKS knockout, a targeting vector for cre-mediated deletion of exon 2 of *Baiap2l1* was generated from the C57BL/6J RPCIB-731 BAC library, and targeted into C57BL/6NTac ES cells. Generation of floxed animals was carried out by Taconic Artemis. IRTKS^*−*/*−*^ mice were obtained by mating B6-Baiap2l1^f/f^ with B6-CMV-Cre. To obtain IRSp53^+/−^ and IRSp53^*−*/*−*^ mice in IRTKS^*−*/*−*^ background, a IRSp53^*−*/*−*^ female was first crossed to a IRTKS^*−*/*−*^ male. The double heterozygous IRSp53^+/−^;IRTKS^+/−^ offspring were intercrossed to obtain the IRSp53^+/−^;IRTKS^*−*/*−*^ mice for experiments. Genotyping of IRSp53^*−*/*−*^ mice was performed using primer pairs: 5′-CACCTTCGCTGCCAAAGGCTA-3′ and 5′-GCACATCTACCCGGGGACC-3′ (IRSp53^+/+^ or 5′-ATCCTCTGCATGGTCAGGTC-3′ and 5′-CGTGGCCTGATTCATTCC-3′ (beta-geo). The site of deletion at exon 2 of the *Baiap2l1* gene was determined by genotyping PCR using 3 primers: 5′-TTTCCTAATGCTGGAGTGATGG-3′, 5′-AGTCCAGGGTTGAATTGTTCC-3′ (WT); 5′-GCTAAGAGAACGATTCTCATGTAGC-3′ (KO). All animal work was carried out in accordance to approved IACUC protocols at the Biological Resource Center, A*STAR.

### Histology, immunohistochemistry and RNA in situ hybridisation

Embryos and placentas were dissected in cold PBS, and fixed in 4% PFA at 4 °C overnight. Paraffin-embedded tissues were sectioned to 5μ thickness and stained with Hematoxylin and Eosin. Immunohistochemistry was performed on the sections by antigen retrieval at pH9 (IRSp53, CD31) and pH6 (IRTKS) in pressure cooker for 1 hr, followed by blocking with 10% goat serum according to standard protocols. The antibodies used: anti-IRSp53 (1:100, Sigma-Aldrich #HPA023310), anti-CD31 (1:25, Abcam #Ab28364) and anti-IRTKS (1:500, Sigma-Aldrich # HPA019484). ISH for *Tpbpa* was done on PFA-fixed paraffin-embedded placental sections using RNAscope probes, following manufacturer’s instructions with the 2.0HD brown kit (Advanced Cell Diagnostics). To detect alkaline phosphate activity, freshly isolated placentas were dissected in cold PBS and cryo-embedded in Frozen Section media (FSC 22, Leica Biosystems). 7 μm sections were fixed in acetone for 5 minutes, air-dried and stained with NBT/BCIP solution, and counterstained with eosin.

### Quantitative analysis of vasculogenesis in placental labyrinth and labyrinth thickness

Hemisected E14.5 placentas were imaged with a Zeiss AxioImager and processed for vessel density and lumen area in ImageJ software. Three placentas for each genotype were analysed. To quantify vasculogenesis, 5 images per placenta immunostained for CD31 were taken under 20x objective. Vessel density was calculated by tracing each CD31^+^ vessel in the field of view, and presented as total number per mm^2^. Vessel lumen area is determined by calculating the median area of all CD31^+^ vessels for each placenta. Labyrinth thickness is defined as the maximum perpendicular distance between the outer labyrinth-spongiotrophoblast border and the inner chorionic plate under H&E staining. It is calculated by taking the average of five readings per placenta, and expressing it as a percentage of the total placental thickness. Three to five placentas for each genotype were analyzed.

### Angiogenesis assay

E14.5 umbilical arteries were cut into 1–3 mm sections and embedded into 1 mg/ml collagen gel (BD Biosciences) in 8-well glass-bottom chamber slides (NUNC). The explants were cultured in complete EGM2-MV media containing 5% fetal bovine serum and the Single-Quot^®^ growth factor/antibiotic cocktail (Lonza), in 5% CO_2_ and 5% O_2_ for 4 days. Explant outgrowths were fixed in 4% PFA at 4 °C for 1 h and stained with anti-CD31, followed by Alexa488-conjugated goat anti-rabbit IgG secondary antibodies and Hoechst (Molecular Probes). Imaging was performed with the Olympus FV1000 inverted confocal laser scanning microscope. Z-stack images were collected every 5–10 microns and maximum projection images were analysed with ImageJ.

### RNA isolation and qPCR

E14.5 placentas were dissected in cold RNAlater solution (Ambion). Total RNA was extracted by homogenizing in Trizol (Invitrogen) using the Tissuelyser (Qiagen) and column purified with RNeasy kits (Qiagen). cDNA was generated from 1 ug total RNA using the High Capacity cDNA Archive kit (Applied Biosystems). Quantitative PCR was carried out using specific primers with PowerSYBR master mix on the ABI 7900HT system. Primer sequences are provided in Supplemental Information.

### Skeleton staining

Embryos were de-skinned, eviscerated and fixed in 95% ethanol for 2 days. The skeletons were stained with Alcian Blue solution (0.03% Alcian Blue, 80% ethanol, 20% acetic acid) for 72 h, followed by washing in 95% ethanol for 24 h, and 95% ethanol containing 2% KOH for 48 h. The skeletons were further stained with Alizarin Red solution (0.03% Alizarin Red, 1% KOH) for 48 h, then cleared in 1% KOH/20% glycerine solution.

### Western Blotting

Total protein lysates were prepared from tissues by bead-beating using a Mikro-dismembrator (Sartorius) followed by solubilisation in RIPA buffer (25 mM Tris-HCl pH 7.6, 150 mM NaCl, 1% NP-40, 1% sodium deoxycholate, 0.1% SDS, Life Technologies), or from cells by direct solubilisation in the same buffer. Twenty-seven μg of protein per lane was loaded and resolved on a 10% SDS-PAGE gel, then semi-dry transferred onto a PVDF membrane. Primary antibodies used were rabbit polyclonal anti-IRSp53 (1:3500), rabbit polyclonal anti-IRTKS (1:3500) and HRP-conjugated mouse monoclonal anti-GAPDH (1:2000). The HRP-conjugated goat anti-rabbit IgG (1: 5000, Santa Cruz Biotechnology #sc-2004) was used as the secondary antibody. Signals were developed by enhanced chemiluminescence using the Supersignal West Femto maximum sensitivity substrate (Thermo scientific).

### Whole-mount IHC staining

Embryos were harvested between E8.5 to E11.5, dissected in cold PBS, and fixed in 4% paraformaldehyde (PFA) in PBS at 4 °C between 2 to 3 h with gentle agitation. Embryos were washed 3 times in PBS containing 1% Triton X-100 at 30 min each time, and blocked in PBS containing 1% Triton X-100 and 10% goat serum at room temperature for 1 h. Embryos were then incubated with rabbit anti-IRSp53 (1:100), followed by HRP-conjugated goat anti-rabbit IgG (1:500), both overnight at 4 °C. Embryos were washed 6 times at room temperature after each antibody incubation, 30 min each time. DAB substrate (DAKO) was added until sufficient coloration has developed.

### Whole-mount beta-gal staining

Embryos were dissected and rinsed in PBS, and fixed for 20 min in 0.3% glutaraldehyde at room temperature. Embryos were then washed thrice in detergent rinse (2 mM MgCl_2_, 0.01% sodium deoxycholate, 0.02% Nonidet P-40, 100 mM sodium phosphate, pH 7.3), 20 min each at room temperature. Beta-gal staining was performed in Staining Solution (2 mM MgCl_2_, 0.01% sodium deoxycholate, 0.02% Nonidet P-40, 100 mM sodium phosphate, pH 7.3, 5 mM potassium ferricyanide, 5 mM potassium ferrocyanide and 1 mg/ml X-gal) for 24 h at room temperature or 30 °C. Embryos were washed in PBS and post-fixed in 4% PFA at 4 °C overnight.

## Additional Information

**How to cite this article**: Chou, A. M. *et al*. Redundant functions of I-BAR family members, IRSp53 and IRTKS, are essential for embryonic development. *Sci. Rep.* **7**, 40485; doi: 10.1038/srep40485 (2017).

**Publisher's note:** Springer Nature remains neutral with regard to jurisdictional claims in published maps and institutional affiliations.

## Supplementary Material

Supplementary Information
