# Supplementary material for: Redundant functions of I-BAR family members, IRSp53 and IRTKS, are essential for embryonic development
Source: Sci Rep. 2017 Jan 9;7:40485. doi: 10.1038/srep40485 (PMC5220365; doi:10.1038/srep40485)
Supplement: Supplementary Information [file srep40485-s1.pdf]

Redundant functions of I-BAR family members, IRSp53 and IRTKS, are essential for embryonic development

Ai Mei Chou[1], Kai Ping Sem[1,#], Wei Jun Lam[2], Sohail Ahmed[1,*], Chin Yan Lim[2,*]
[1]Neural Stem Cells Laboratory, Institute of Medical Biology, Singapore 138648
[2]Epithelial Epigenetics and Development Laboratory, Institute of Medical Biology, Singapore 138648
[#]Present address: Translational Laboratory in Genetic Medicine, Singapore 138648
*Corresponding authors

Contact:
Chin Yan Lim: chinyan.lim@imb.a-star.edu.sg
Sohail Ahmed: sohail.ahmed@imb.a-star.edu.sg

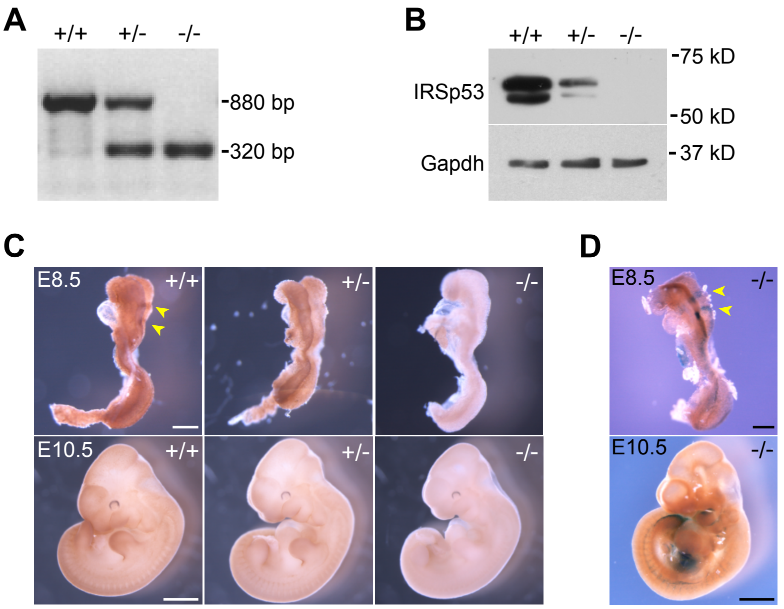

Figure S1. IRSp53 expression in embryos. (**A**) Genotyping of IRSp53 wild-type, heterozygous and knockout embryos. Gel image was cropped for clarity. Full-length gel is presented in Fig S5B. (**B**) Western blot showing IRSp53 levels in brain tissue lysates of adult mice. Blots were cropped and processed for clarity. Full length blots are presented in Fig S5C. (**C**) Whole-mount immunostaining of IRSp53 in E8.5 and E10.5 embryos. IRSp53 was widely detected at E8.5 with elevated levels in specific rhombomeres (arrowheads). Scale: 100μm. At E10.5, expression was stronger in the branchial arches, limb buds and somites. Scale: 500 μm. (**D**) Expression from the IRSp53-lacZ reporter allele in IRSp53-/- embryos at E8.5 and E10.5 detected by lacZ staining. Expression, particularly in the rhombomeres, corresponds to endogenous IRSp53 expression. Scale bars: 100 μm (E8.5), 500 μm (E10.5).

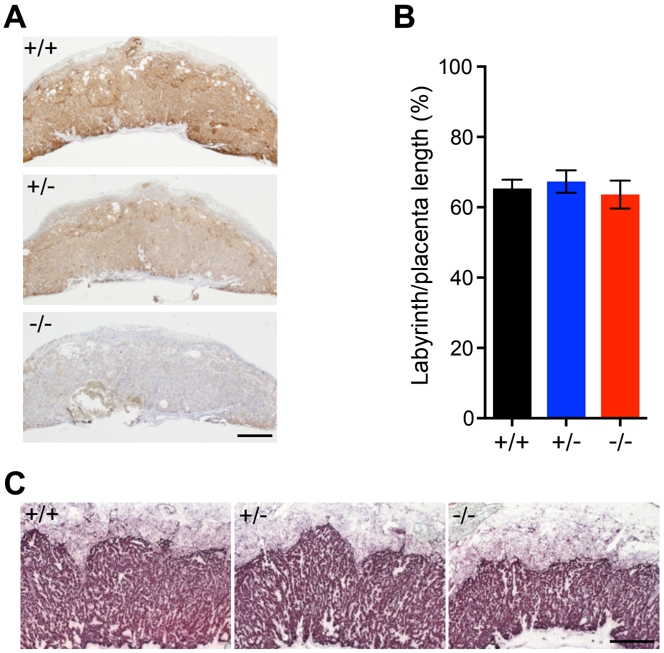

Figure S2. IRSp53 expression in placenta. (**A**) High levels of IRSp53 in wildtype E14.5 placenta detected by immunostaining. Scale bar: 500 μm. (**B**) The labyrinth thickness of IRSp53 wild-type, heterozygous and null placenta were measured. Images of 3 placentas were analyzed for each genotype. Error bars indicate s.d. (**C**) Organization of maternal blood spaces in the labyrinth of E14.5 placentas revealed by intrinsic alkaline phosphatase activity in trophoblast cells lining the lumen. Scale bar: 500 μm.

A

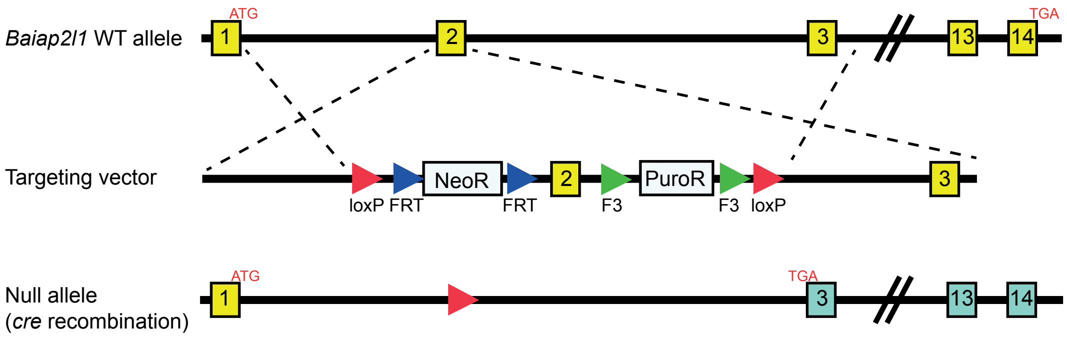

B

```
CLUSTAL multiple sequence alignment by MUSCLE (3.8)


IRSp53|NP_001032843.1      MSLSRSEEMHRLTENVYKTIMEQFNPSLRNFIAMGKNYEKALAGVTFAAKGYFDALVKMG
IRTKS|NP_080109.1          MSRG-PEEVNRLTENTYRNVMEQFNPGLRNLINLGKNYEKAVNAMILAGKAYYDGVAKIG
                           ** . .**::*****.*..:******.***:* :*******: .: :*.*.*:*.:.*:*

IRSp53|NP_001032843.1      ELASESQGSKELGDVLFQMAEVHRQIQNQLEETLKSFHNELLTQLEQKVELDSRYLSAAL
IRTKS|NP_080109.1          EIATGSPVSTELGHVLIEISSTHKKLNETLDENFKKFHKDIIHELEKKTELDVKYMNATL
                           *:*: *  *.*** **::::..*.:::: *:*.:*.**:::: :**:*.*** .*:.*:*

IRSp53|NP_001032843.1      KKYQTEQRSKGDALDKCQAELKKLRKKSQGSKNPQKYSDKELQYIDAISNKQGELENYVS
IRTKS|NP_080109.1          KRYQAEHRNKLDSLEKSQAELKKIRRKSQGGRNALKYEHKEIEYVETVTSRQSEIQKFIA
                           *.**:*:*.* *:*:*.******:*.****..*. **. **::*:::::..*.*::::::

IRSp53|NP_001032843.1      DGYKTALTEERRRFCFLVEKQCAVAKNSAAYHSKGKELLAQKLPLWQQACADPNKIPDRA
IRTKS|NP_080109.1          DGCKEALLEEKRRFCFLVDKHCSFASHIHYYHMQSAELLNSKLPRWQETCCDATKVPEKI
                           ** * ** **.*******:*:*:.*.:   ** :. *** .*** **::*.*..*:*:.

IRSp53|NP_001032843.1      VQLMQQMANS-----NGSILPSALSASKSNLVISDPIPGAKPLPVPPELAPFVGSQSKLS
IRTKS|NP_080109.1          MNMIEEIKTPISTPVSGTPQPSPM-IERSKMIGKDYDTLSKYSPKMPPAPSVKAYTSPLI
                           ::::::: ..     .*:  **.:  ..*::: .*  . :*  *  *  ... .  * *

IRSp53|NP_001032843.1      DSYSNTLPVRKSVTPKNSYA--TTENKTLPRSSSMAAGLERNGRMRVKAIFSHAAGDNST
IRTKS|NP_080109.1          DMFNNPATAAQSSEKTNNSTANTGEDPSLQRSVSVATGLNMMKKQKVKTIFPHTAGNNKT
                           * :.*. .. :*   .*. :  * *: :* ** *:*:**:   . .**:**.*:**:*.*

IRSp53|NP_001032843.1      LLSFKEGDLITLLVPEARDGWHYGESEKTKMRGWFPFSYTRVLDSDGSDRLHM-SLQQGK
IRTKS|NP_080109.1          LLSFAQGDVLTLLIPEEKDGWLYGEHDTTKARGWFPSSYTKLLEENEAMSVPTPSPAPVR
                           **** :**::***:** .*** *** :.** ***** ***.:*:.: :  :   *    .

IRSp53|NP_001032843.1      SSSTGNLLDKDDLALPPPDY-------GTSSR---AFPTQTAGTFK---QRP--------
IRTKS|NP_080109.1          SISTVDLTEKSSVVIPPPDYLECLSMGATSDKKAGAPKVPSASTFRAPVSRPDATSTSPS
                           * ** :* :*..:.:*****       .**..   *  . :*.**.   .**

IRSp53|NP_001032843.1      --YSVAVPAFSQGLDDYGA----RSVSSGSGTLVSTV
IRTKS|NP_080109.1          DANGTAKPPFLSGENPFATVKLRPTVTNDRSAPIIR-
                              ..* *.* .* : :.:     :*:.. .: :
```

Figure S3. IRTKS may exhibit functional redundancy with IRSp53. (**A**) Conditional knockout strategy to delete exon 2 of the Baiap2L1 (IRTKS) gene. The null allele results in splicing of exon 1 to exon 3, which is expected to create a premature stop codon in exon 3 and give a 18-amino acid truncated product that lacks all functional domains. (**B**) Protein sequence alignment of mouse IRSp53 and IRTKS. The two shares the highest identity (42%) among I-BAR domain family members with the most sequence conservation at the N-terminal region.

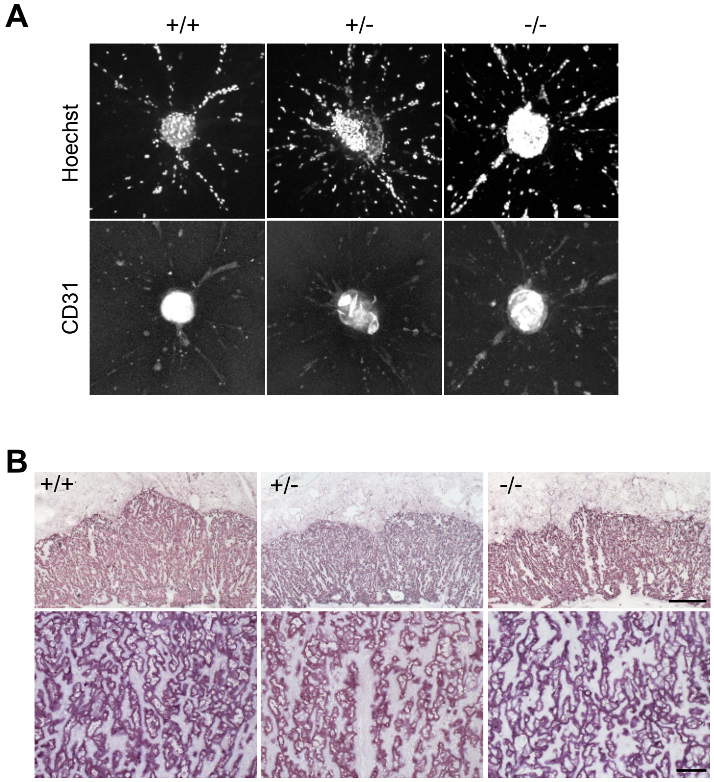

Figure S4. Loss of IRSp53 and IRTKS do not affect vascularization. (**A**) In vitro microvessel formation from E14.5 umbilical cord sections examined by staining explants cultured for 4 days in collagen I gels with Hoescht and CD31 antibodies. Explants were homozygous null for IRTKS and wild-type, heterozygous or null for IRSp53. (**B**) Organization of maternal blood spaces in the labyrinth of E14.5 placentas revealed by intrinsic alkaline phosphatase activity in trophoblast cells lining the lumen (upper panels). Lower panels show the alkaline phosphatase-positive trophoblast cells lining the maternal sinusoids at higher magnification. Scale: 500 μm (upper panels); 100 μm (lower panels).

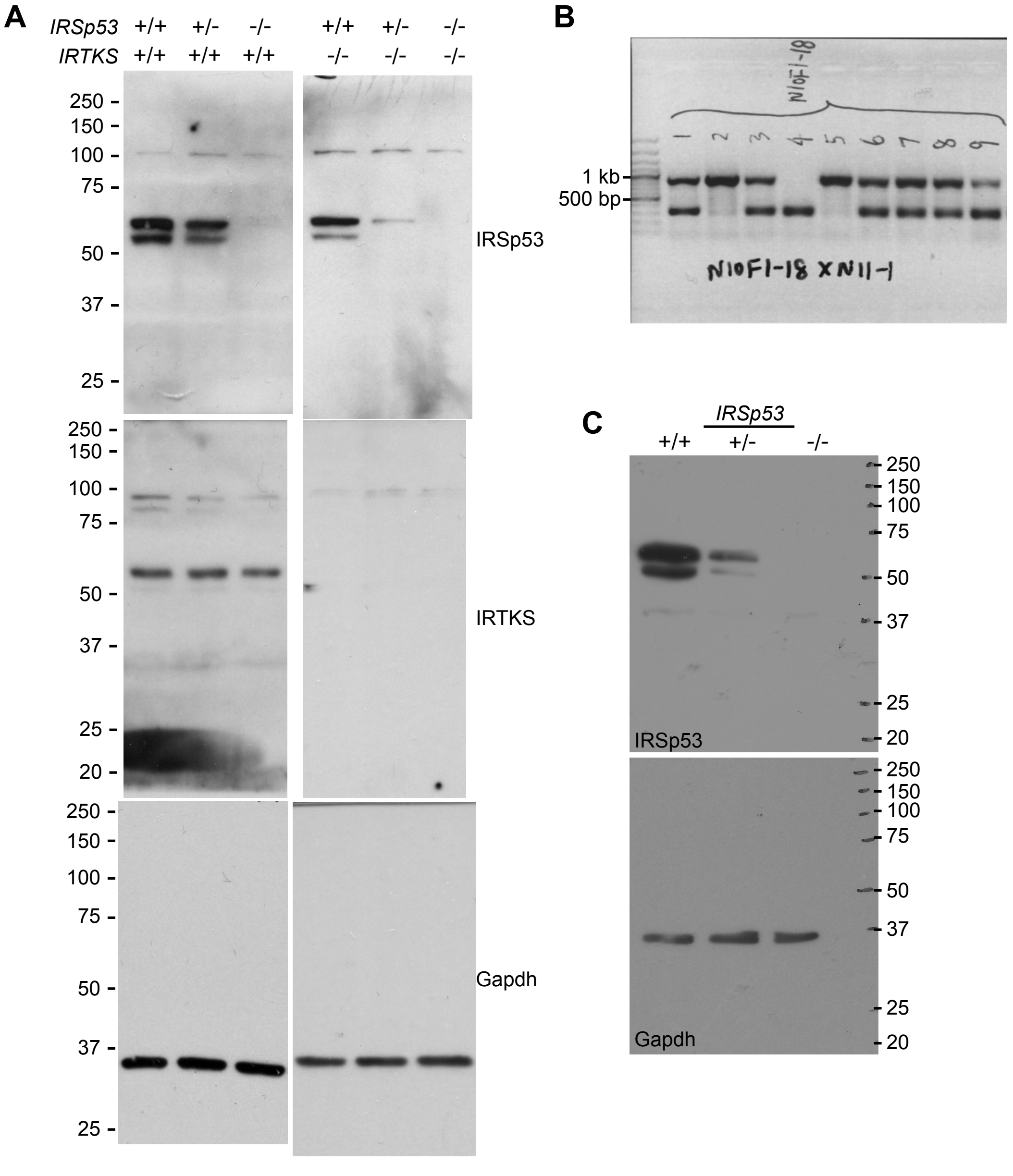

Figure S5. Full-length blots and gels. (**A**) Full-length blots of cropped images shown in Fig 4A (**B**) Full-length DNA gel showing genotyping of embryos from IRSp53+/- intercross. Lanes 2-4 were cropped and presented in Fig S1A (**C**) Full-length western blots of cropped images shown in Fig S1B.

Table 1. Primer Sequences for qPCR

Gene Name	Forward Primer	Reverse Primer
Ascl2	GAGAGCTAAGCCCGATGGA	AGGTCCACCAGGAGTCACC
Cav1	CAACGACGACGTGGTCAAG	CACAGTGAAGGTGGTGAAGC
Cdx2	CACCATCAGGAGGAAAAGTGA	CTGCGGTTCTGAAACCAAAT
Ednrb	TCAGAAAACAGCCTTCATGC	GCGGCAAGCAGAAGTAGAA
Esx1	CGCTACCGCATCTGCTTC	CTGCACTCTAGGTTCAGGTAAGC
Flt1	GACGGTCATAGAAGGAACAAATAAG	CCCGGCAGCTGTAGATTC
Gcm1	GAGGCACGACGGACGCT	TCTCCGGCCTGGGATGA
Hand1	CAAGCGGAAAAGGGAGTTG	GTGCGCCCTTTAATCCTCTT
Id1	GCGAGATCAGTGCCTTGG	CTCCTGAAGGGCTGGAGTC
IRSp53	TCTGCTGCTTACCATTCCAA	GCACGGTCTGGGATCTTG
Lyve1	GGTGTCCTGATTTGGAATGC	ATGCAGGAGTTAACCCAGGTG
Pecam1	CGGTGTTCAGCGAGATCC	ACTCGACAGGATGGAAATCAC
Prl2c2	CAAGCCAGGCTCACACACTA	GATCGTCCAGAGGGCTTTCC
Prl3d1	TCTGGAGGGACTTCAGACAATATACA	TGACTGCAGTTCTTCGAGTCCA
Stra13	CAGCGGGGATGCACTGCAGC	CAGCTGATCCACTTCCACAA
Tbp	GGGAGCTGTGATGTGAAGTTC	CCAGGAAATAATTCTGGCTCA
Tpbpa	GACCTCTGAAGAGCTGAACCA	CCAGGCATAGGATGACTAGGA
